# Azimuthal invariance to looming stimuli in the *Drosophila* giant fiber escape circuit

**DOI:** 10.1242/jeb.244790

**Published:** 2023-04-20

**Authors:** HyoJong Jang, David P. Goodman, Jessica Ausborn, Catherine R. von Reyn

**Affiliations:** ^1^ Drexel University, School of Biomedical Engineering, Science and Health Systems, Philadelphia, PA 19104, USA; ^2^ Drexel University College of Medicine, Department of Neurobiology and Anatomy, Philadelphia, PA 19129, USA

**Keywords:** Bilateral integration, Electrophysiology, Visual feature integration, Visual system, Descending neuron, Sensorimotor

## Abstract

Spatially invariant feature detection is a property of many visual systems that rely on visual information provided by two eyes. However, how information across both eyes is integrated for invariant feature detection is not fully understood. Here, we investigated spatial invariance of looming responses in descending neurons (DNs) of *Drosophila melanogaster.* We found that multiple looming responsive DNs integrate looming information across both eyes, even though their dendrites are restricted to a single visual hemisphere. One DN, the giant fiber (GF), responds invariantly to looming stimuli across tested azimuthal locations. We confirmed visual information propagates to the GF from the contralateral eye, through an unidentified pathway, and demonstrated that the absence of this pathway alters GF responses to looming stimuli presented to the ipsilateral eye. Our data highlight a role for bilateral visual integration in generating consistent, looming-evoked escape responses that are robust across different stimulus locations and parameters.

## Teaser

**Editor's choice:** *Drosophila* descending neurons respond to visual looming information from the contralateral eye, even though their dendrites are ipsilateral, highlighting the importance of bilateral visual integration in generating location-invariant escape responses.

## INTRODUCTION

Animals utilize or ignore the positional information of a visual stimulus when selecting behaviors (Allen et al., 2021; Benucci, 2022; Bianco et al., 2011; DiCarlo et al., 2012; Gancedo et al., 2020; Peek, 2018; Pouget and Snyder, 2000; Rolls, 2012). If an object approaches on a direct collision course, its location can inform the selection of motor programs, including the fleeing direction of crabs and mice, postural adjustments preceding takeoff in *Drosophila melanogaster* and C-start direction in fish (Dunn et al., 2016; Hemmi and Tomsic, 2012; Peek, 2018). Other motor programs appear to be location invariant, including freezing behaviors and the leg extension and wing depression of the takeoff jump of *Drosophila* (Card and Dickinson, 2008; von Reyn et al., 2014; Zacarias et al., 2018).

With the exception of the directional C-start responses (Domenici and Hale, 2019), we know little about circuit mechanisms resolving the location of an object approaching on a direct collision course in animals whose visual world is assembled across two eyes. Neurons that respond to looming stimuli (the 2D projection of an object approaching at a constant velocity) have been identified across species (Dunn et al., 2016; Gabbiani et al., 1999; Liu et al., 2011; Oliva and Tomsic, 2014; Sun and Frost, 1998; von Reyn et al., 2014), and receptive fields have been mapped for looming responsive retinal ganglion cells (RGCs) and visual projection neurons (VPNs) in the vertebrate retina and invertebrate optic lobe, respectively (Kerschensteiner, 2022; Klapoetke et al., 2022; Morimoto et al., 2020). But investigations studying where information from the two eyes is combined in the brain have been limited (Gabbiani et al., 2001; Isa et al., 2021; Judge and Rind, 1997; Lee et al., 2020; Rosner and Homberg, 2013; Sun and Frost, 1998; Temizer et al., 2015; Zhao et al., 2014). In *Drosophila*, this is predicted to first occur in the optic glomeruli where descending neurons (DNs) receive information from VPNs before descending into the ventral nerve cord to synapse onto motor and premotor circuits (Namiki et al., 2018; Wu et al., 2016).

Here, we investigated location-dependent tuning in DNs predicted to participate in looming-evoked behaviors. We surprisingly found that multiple looming responsive DNs have significant bilateral responses to looming stimuli even though their dendrites are unilateral. Focusing on one DN, the giant fiber (GF), we found invariant responses across azimuthal approach directions. By occluding one eye with paint, we demonstrated that visual information propagates from the contralateral eye through an unidentified pathway, and that this information is key for the GF response to be maintained regardless of the approach direction. We also found integration across the two eyes is necessary for maintaining the GF response across loom speeds.

## MATERIALS AND METHODS

### Fly stocks

*Drosophila melanogaster* were raised on standard cornmeal fly food at 25°C and 60% humidity on a 12 h light:dark cycle. Female *Drosophila*, because of their larger size in comparison with males, were used for all electrophysiology, anatomy and behavioral experiments 2–5 days after eclosion. The following fly stocks were used for the experiments: (1) GF-split-GAL4: *68A06_AD (attP40); 17A04_DBD (attP2)* (von Reyn et al., 2014), (2) DNp02-split-GAL4: *VT063736_AD (attP40); VT017647_DBD (attP2)* (Namiki et al., 2018), (3) DNp03-split-GAL4: *R30C12_AD (attP40); R22D06_DBD (attP2)* (Namiki et al., 2018), (4) DNp06-split-Gal4: *VT019018_AD (attP40); VT017411_DBD (attP2)* (Namiki et al., 2018) and (5) *w+ UAS-GFP* (Pfeiffer et al., 2010).

### Electrophysiology

*In vivo* whole-cell, current-clamp electrophysiology was carried out on behaving, tethered flies as previously described (von Reyn et al., 2017). Flies were anesthetized at 4°C and their head and thorax were tethered to polyether ether ketone (PEEK) plates with UV glue (Loctite 3972). The T1 legs were cut at the femur to avoid cleaning of the head and occlusion of the eyes. The proboscis was glued in its retracted position to decrease brain movement during the recording. The antennae were also immobilized with UV glue to limit stimulation of antennal afferents to the GF. For eye painting experiments, black paint (Golden High Flow Paint, carbon black acrylic) was applied to one eye. To access the GF for recordings, the cuticle and trachea on the posterior side of the head above the GF soma were removed and the brain was perfused with standard extracellular saline (NaCl 103 mmol l^−1^, KCl 3 mmol l^−1^, TES 5 mmol l^−1^, trehalose·2H_2_O 8 mmol l^−1^, glucose 10 mmol l^−1^, NaHCO_3_ 26 mmol l^−1^, NaH_2_PO_4_ 1 mmol l^−1^, CaCl_2_·2H_2_O 1.5 mmol l^−1^ and MgCl_2_·6H_2_O 4 mmol l^−1^; Gouwens and Wilson, 2009). Osmolarity was adjusted to 270–275 mOsm and bubbled with 95% O_2_/5% CO_2_ to maintain a pH of 7.3. All experiments were performed at room temperature (20–22°C). A brief, localized application of collagenase (0.5% in extracellular saline) with a glass electrode was used to disrupt the brain sheath and access the soma for recording. GFP-labeled somata were then targeted by patch-clamp electrodes (3–6 MΩ) containing intracellular saline (potassium aspartate 140 mmol l^−1^, KCl 1 mmol l^−1^, Hepes 10 mmol l^−1^, EGTA 1 mmol l^−1^, Na_3_GTP 0.5 mmol l^−1^, MgATP 4 mmol l^−1^, Alexafluor-568 5 µmol l^−1^, 265 mOsm, pH 7.3). *In vivo* whole-cell recordings were conducted in current-clamp mode using a MultiClamp 700B amplifier, and digitized (NI-DAQ, National Instruments) at 20 kHz. All data were acquired using the open-source software Wavesurfer (https://wavesurfer.janelia.org/) running in MATLAB (MathWorks). Traces were not corrected for a 13 mV liquid junction potential (Gouwens and Wilson, 2009). Recordings were considered acceptable when the initial seal resistance was >2 GΩ before rupture, the resting membrane potential was less than −50 mV, and the input resistance was >50 MΩ, as the typical input resistance of the GF has been reported to be in the range of 50 to 100 MΩ (Lehnert et al., 2013; von Reyn et al., 2014).

### Visual stimuli for electrophysiology

**Fig. 1.**
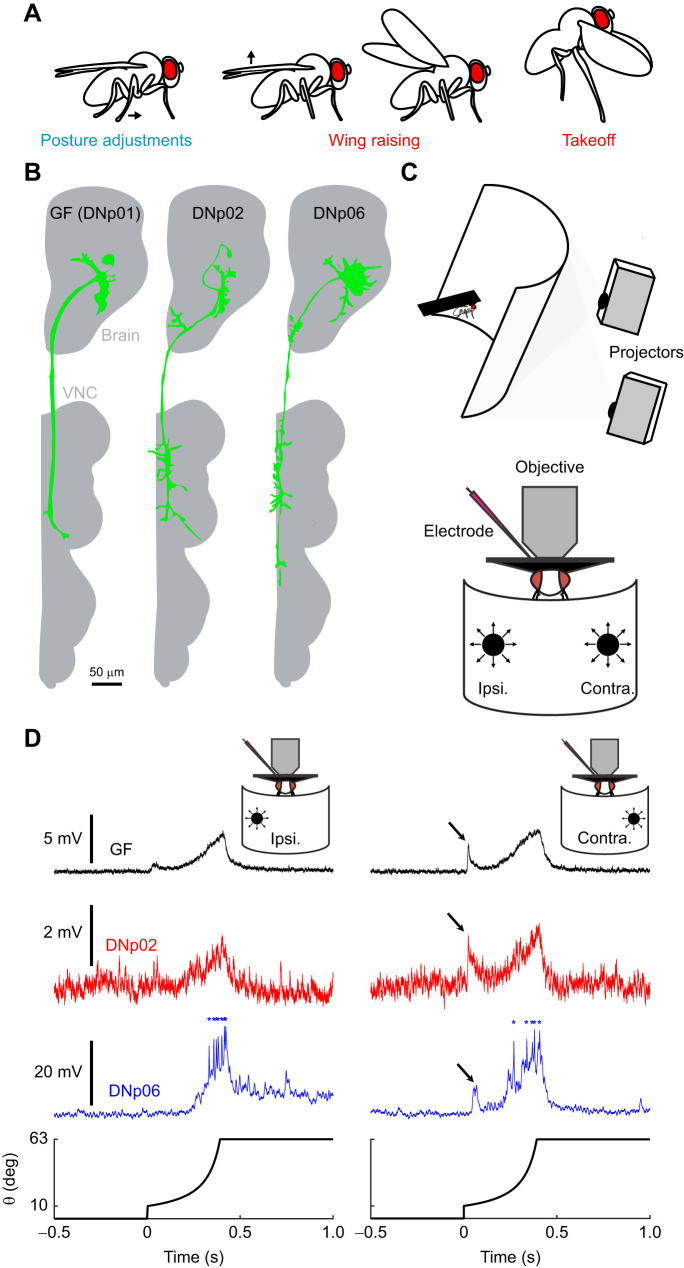
**Looming responsive descending neurons (DNs) integrate looming information both ipsilaterally and contralaterally to their dendrite locations.** (A) Escape motor programs (Card and Dickinson, 2008). (B) DN anatomy. GF, giant fiber; VNC, ventral nerve cord. (C) Preparation for whole-cell electrophysiology during looming stimuli presentations. Ipsi., ipsilateral; Contra., contralateral. (D) DN ipsilateral and contralateral looming responses. GF and DNp02 traces are response averages (*n*=10 and 2, respectively), DNp06 trace is from a single trial; asterisks indicate spikes. Bottom trace, angular size (θ) of the looming stimulus over time (radius to approach speed ratio *r*/*v*=40 ms). Arrows indicate ‘pop-on’ responses to the appearance of the stimulus on the screen.

Visual stimuli were back-projected onto a 4.5 inch diameter mylar cylindrical screen covering 180 deg in azimuth via two DLP projectors (Texas Instruments Lightcrafter 4500). To project onto a cylindrical surface, each projector was calibrated as described previously (Goodman et al., 2018) and an 18 deg overlap between the two projectors was blended for uniform illumination (Fig. 1C). Following calibration and blending, each 912×1140 resolution projected image was displayed in 6 bit grayscale at 240 Hz, above the flicker fusion frequency of *Drosophila* (100 Hz; Niven et al., 2003). All stimulus frames were generated in MATLAB and presented using psychtoolbox (http://psychtoolbox.org/; Brainard and Vision, 1997).

Looming stimuli were generated with the background set to white and the expanding disk (looming stimulus) set to black on the projector (5500 lx-white and 15 lx-black, measured at the position of the fly). The angular size (θ) of the stimulus subtended by the approaching object was calculated in time (*t*) by the following equation (Gabbiani et al., 1999):
(1)

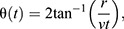
where *t*<0 before collision and *t=*0 at collision for an approaching object with a radius (*r*) and constant velocity (*v*). As described previously (Gabbiani et al., 1999), *r*/*v* (ms) is the radius to approach speed ratio. The smaller the *r*/*v*, the more abrupt the stimulus expansion.

The visual stimulus set consisted of (1) four looming stimuli (*r*/*v=*10, 20, 40, 80 ms) starting at 10 deg and expanding to 63 deg in angular size and held for 1 s, displayed at three locations (−45, 0, 45 deg in azimuth, 0 deg in elevation); (2) three constant angular velocity stimuli (100, 2000, 6000 deg s^−1^) starting at 10 deg and expanding to 63 deg and held for 1 s, displayed at the three azimuthal locations; (3) small object (10 deg disk) motion stimuli starting and ending at three locations in azimuth (−67.5 deg to −22.5 deg, −22.5 deg to 22.5 deg, 22.5 deg to 67.5 deg) moving at 22.5 deg s^−1^ from left to right; and (4) dual looming stimuli (*r*/*v=*10, 20, 40, 80 ms) approaching simultaneously from 45 deg and −45 deg in azimuth and 0 deg in elevation. Each visual stimulus was presented once per trial, in a randomized order. Interstimulus intervals were set to 30 s to provide enough time for the GF membrane potential to return to baseline and avoid habituation. The responses to the same stimulus from two successive trials were then averaged for each fly.

### Direction selectivity

A direction selectivity index (DSI) (Elstrott et al., 2008; Hei et al., 2014) was calculated for each fly across stimulus locations to see whether the GF in individual flies was more selective to stimuli presented to the eye ipsilateral to its dendrites (−45 deg) or contralateral to its dendrites (45 deg), or to ipsilateral stimuli over frontal (0 deg) stimuli that expand across both eyes:
(2)

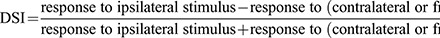
To evaluate whether DSI scores indicated significant direction selectivity, a permutation test (Baden et al., 2016) was performed by generating a null hypothesis distribution (10,000 shuffled trials for responses between the two directions) representing no directional bias. The *P*-value was then computed by calculating the percentage of the null distribution above the mean of the group DSI.

### Data analysis and statistics

All analyses were performed using in-house MATLAB scripts. Electrophysiology recordings for each stimulus presentation were baseline subtracted by taking the average response 1 s preceding the stimulus onset. The GF expansion peak magnitude and latency following stimulus onset were measured after filtering each recording (Savitzky–Golay, fourth order polynomial, frame size is 1/10th the length of the stimulus). The GF ‘pop-on peak’, a short-latency, transient depolarization that occurs when the stimulus first appears on the screen, was measured in magnitude and latency within a 50 ms time window after the onset of the stimulus. To select the appropriate parametric or non-parametric test, the Shapiro–Wilk test was used to evaluate whether data were normally distributed. We found our data were normally distributed and therefore used an ANOVA and Tukey's HSD *post hoc* test except in the calculation of the DSI, as discussed above. We also performed a power analysis (significance criterion of alpha=0.05 and power=0.90) to evaluate our ability to detect effects, given our sample size (Table S1).

All data and software generated for this paper are available from the corresponding author upon request.

## RESULTS AND DISCUSSION

For *Drosophila*, a looming stimulus's location impacts the selection of certain motor programs; for example, the postural adjustments needed to establish takeoff trajectory (Card and Dickinson, 2008; Peek, 2018) (Fig. 1A). Other motor programs, such as freezing or takeoff leg extension and wing depression, seem invariant to stimulus direction (Card and Dickinson, 2008; von Reyn et al., 2014; Zacarias et al., 2018). Motor program selection is thought to happen at the level of DNs that integrate sensory information in the brain and activate motor circuits in the ventral nerve cord (VNC). The GF (aka DNp01) is the only DN that has a well-established role, driving leg extension and wing depression for takeoffs (Ache et al., 2019; Allen et al., 2006; von Reyn et al., 2014, 2017). However, recent anatomical, functional and behavioral evidence suggest additional DNs (including DNp02 and DNp06; Fig. 1B) drive other motor programs or takeoffs in the absence of GF activation (Fotowat et al., 2009; Namiki et al., 2018; Peek, 2018; von Reyn et al., 2014; Zacarias et al., 2018).

We investigated directional selectivity of looming responses in the GF, DNp02 and DNp06 (Fig. 1B) (Namiki et al., 2018; Peek, 2018). These DNs exist as bilateral pairs with dendrites restricted to the hemisphere ipsilateral to their soma (Namiki et al., 2018). This anatomy suggests each DN responds preferentially to looming stimuli presented to the ipsilateral eye, with contralateral looming stimuli encoded by its contralateral partner. We modified our previous visual setup (Goodman et al., 2018) to expand coverage of the fly's azimuthal visual field to 180 deg (Fig. 1C). We then recorded, using whole-cell electrophysiology, DN responses to looming stimuli with ipsilateral or contralateral approach directions (±45 deg azimuth). Stimuli were dark disks with an initial angular size of 10 deg that expanded to 63 deg to restrict excitation to one eye (Fig. 1C). Interestingly, we found all DNs responded significantly to both stimuli, despite their unilaterally projecting dendrites (Fig. 1D). We also found, as reported previously (Peek, 2018), that DNs show distinct responses to these stimuli (Fig. 1D). For example, DNp02 responses remained subthreshold while DNp06 showed large depolarizations and spikes during the stimulus expansion. As *Drosophila* escape responses likely require multiple DNs to enable behavioral complexity and flexibility (Namiki et al., 2018; Peek, 2018; von Reyn et al., 2014; Zacarias et al., 2018), including additional DNs that we did not investigate here, the observed differences in DNs' looming responses could represent different motor programs (Card and Dickinson, 2008).

**Fig. 2.**
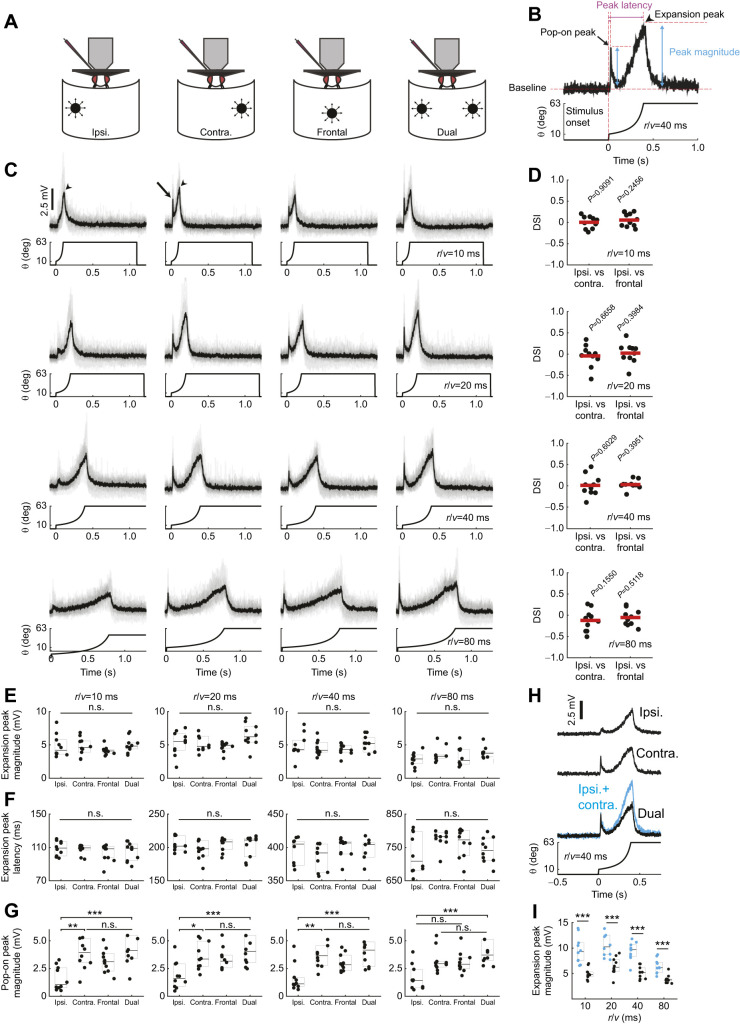
**GF directional invariance to looming stimuli.** (A) Looming stimuli locations used for these investigations. (B) Components of the GF looming response. (C) GF average (black) and individual (gray) responses to looming stimuli across *r*/*v* and locations (indicated in A). (D) Direction selectivity index (DSI) across individual flies (red lines are means; *P*-values as indicated). (E–G) Quantification of C following the measurements in B (E, expansion peak magnitude; F, expansion peak latency; G, pop-on peak magnitude). Box plots show median, and upper and lower quartiles; circles are individual data points (*P<0.05, **P<0.01, ***P<0.001; *n*=10 flies). (H) Sum of an individual fly's response to ipsilateral and contralateral stimuli (blue) compared with the actual response to a dual stimulus (black). (I) Quantification of H across *r*/*v* (*P<0.05, **P<0.01, ***P<0.001; *n*=10 flies).

We next focused our investigation on GF visual integration and added looming presentations from the front of the fly that expanded across both eyes (Fig. 2A, frontal), and concurrent unilateral looming stimuli exciting both eyes (Fig. 2A, dual). We also presented looming stimuli at a range of radius/approach speed (*r*/*v*) values (Gabbiani et al., 1999), where smaller values indicate abrupt expansions.

To compare GF responses, we measured response components as indicated in Fig. 2B. We found the GF peak response magnitude was not significantly different across stimulus locations (Fig. 2C,E), suggesting the GF response is invariant to azimuthal stimulus locations within the evaluated horizontal visual field. We also calculated a DSI to quantify the selectivity of individual flies to ipsilateral over contralateral or frontal stimuli (Fig. 2D). While we found DSIs to be variable across flies, they were not significantly different from a randomized distribution of responses (Fig. 2D), suggesting the GF has minimal to no directional preference across tested stimulus locations.

When both eyes were stimulated with the dual looming stimulus, the GF response magnitude was significantly less than the sum of the individual responses across all *r*/*v* values (Fig. 2H,I). Dual loom responses were also not significantly different from a single loom response (Fig. 2E), suggesting the GF may quickly reach an upper bound on its response through intrinsic or circuit-level mechanisms. This was surprising given that GF ipsilateral visual integration is linear and supralinear (Ache et al., 2019; von Reyn et al., 2017) but similar to sublinear dual loom integration in the locust LGMD/DCMD. However, in locust studies, both looms were restricted to one eye (Guest and Gray, 2006; Krapp and Gabbiani, 2005). Given that the GF and LGMD/DCMD utilize different looming feature integration algorithms (Gabbiani et al., 1999; von Reyn et al., 2017), it would be interesting to compare bilateral integration mechanisms across species.

We next investigated the timing of the peak GF response across stimulus locations, as response timing between looming-sensitive DNs is hypothesized to guide escape behavior selection (Card and Dickinson, 2008; von Reyn et al., 2014). We hypothesized that responses emerging from the contralateral side may be delayed compared with responses from the ipsilateral side through direct connections with VPN outputs from the optic lobe (Ache et al., 2019; von Reyn et al., 2017). However, we found no significant difference in the peak latency (Fig. 2F). These data suggest the GF can consistently time its response regardless of azimuthal approach direction.

While peak response magnitude and timing were invariant across azimuthal stimulus locations, we witnessed a difference in the presence of a transient response that occurs when the looming stimulus first appears on the screen as a 10 deg disk. This ‘pop-on’ transient was present in the response to contralateral looms but reduced in magnitude for ipsilateral looms (Fig. 2C,G). Interestingly, this difference was also observed across other DNs (Fig. 1D arrows). We hypothesize this may be because our visual display illuminates both eyes, as this transient occurs in the GF when visual displays illuminate a single eye (Ache et al., 2019; von Reyn et al., 2017). Prior work has demonstrated LC4 contributes predominantly to the pop-on response in the GF (von Reyn et al., 2017). Our data therefore suggest brightness differences across the eyes may modulate the LC4 contribution to DN responses.

**Fig. 3.**
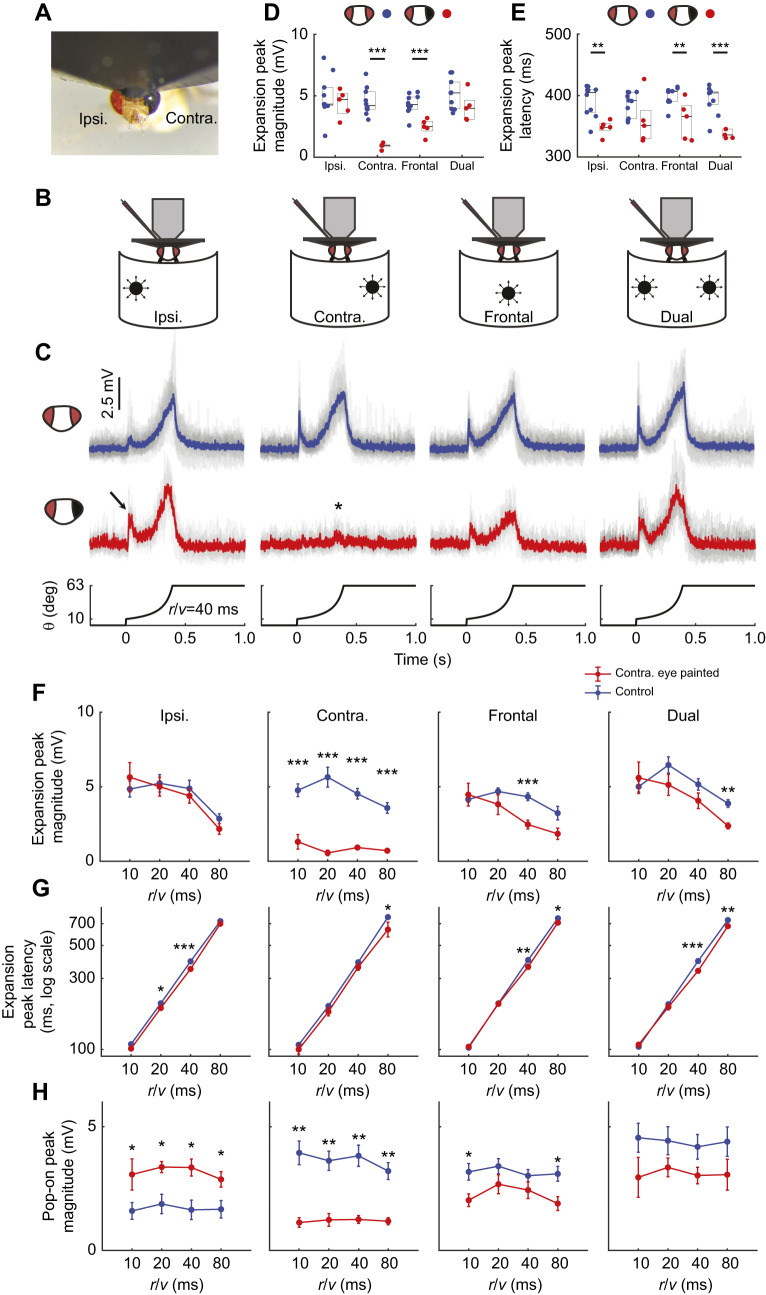
**GF tuning to ipsilateral stimuli is modulated by information from the contralateral eye.** (A) Painted contralateral eye in a tethered fly. (B) Looming stimuli locations used for these investigations. (C) GF individual (gray) responses to looming stimuli (*r*/*v=*40 ms*)* across locations (indicated in B) with (red, average) and without (blue, average) contralateral eye painting. Arrow indicates regained pop-on peak and asterisk indicates the response from ipsilateral eye stimulation. (D,E) Quantification of the data in C (D, expansion peak magnitude; E, expansion peak latency). Box plots as in Fig. 2 (*P<0.05, **P<0.01, ***P<0.001; control *n*=10 flies, painted *n*=5 flies). (F–H) Quantification of eye painting experiments across *r*/*v* (F, expansion peak magnitude; G, expansion peak latency; H, pop-on peak magnitude). Values indicate means±s.e.m. for control (blue) and painted (red) flies (*P<0.05, **P<0.01, ***P<0.001; control *n*=10 flies, painted *n*=5 flies).

As our data suggest the GF receives information from the contralateral eye, we wanted to validate these findings with eye occlusion experiments. We painted the contralateral eye with opaque black paint and displayed our visual stimulus set while recording from the GF. As hypothesized, occlusion of the contralateral eye eliminated the GF response for all but the final expansion of the contralateral looming stimulus (Fig. 3C,D,F). We assume the remaining response marks when the stimulus crosses into the receptive field of the ipsilateral eye (Fig. 3C, asterisk), within the fly's estimated static (Buchner, 1971) or retinal muscle-induced dynamic binocular region (Fenk et al., 2022). As the VPNs LC4 and LPLC2 that provide ipsilateral visual input to the GF have small, ipsilaterally restricted receptive fields (Klapoetke et al., 2022), these data suggest the GF receives contralateral information through an unidentified commissural pathway.

We anticipated contralateral eye painting would have minimal effect on ipsilateral looming stimuli but significantly reduce the response to frontal looms. Indeed, eye painting did not significantly affect GF peak response magnitude to ipsilateral looms. Interestingly, eye painting reduced the response to frontal looms but only for larger *r*/*v* values (Fig. 3D,F), shifting GF tuning preference towards abrupt looms. As LC4's angular velocity contribution to the GF response increases as *r*/*v* decreases (while LPLC2's angular size contribution remains constant; von Reyn et al., 2017), these data again suggest LC4 input to the GF circuit is particularly susceptible to brightness differences across both eyes and that visual information from both eyes establishes GF tuning.

We then investigated how contralateral eye painting affects GF response timing (Fig. 3G). We hypothesized that loss of excitation from the other eye would decrease the peak latency for ipsilateral, frontal and dual looms as they would no longer benefit from the stimulus expanding into the occluded region of the fly's visual field. Indeed, we found shifts to shorter latencies across these loom locations.

Finally, we investigated how contralateral eye painting changes the magnitude of the GF pop-on transient (Fig. 3H). As the starting size of the stimulus does not change across *r*/*v* values, we did not anticipate significant differences in magnitude except a complete loss of the response in the painted contralateral eye. While the contralateral, frontal and dual stimulus results followed our predictions, we unexpectedly regained the pop-on transient for the ipsilateral stimulus. While unexpected, these results support our hypothesis for the reduction of the pop-on peak in control flies: LC4 contributions are modulated by brightness differences across the eye.

In summary, we found a subset of DNs (GF, DNp02, DNp06) respond to looming information from the contralateral eye even though their dendrites are ipsilateral (Namiki et al., 2018). To date, only GF ipsilateral connectivity is well established (Ache et al., 2019; von Reyn et al., 2017) while contralateral connectivity is not known for these DNs. GF axons form electrical synapses in the VNC (Allen et al., 2006); however, the similar latency and magnitude of contralateral and ipsilateral responses suggest contralateral visual information is not arriving through this connection as it would be attenuated and delayed. The GF is also electrically coupled to giant commissural interneurons (aka AMMC-A1) that receive direct information from the fly's auditory system (Pézier et al., 2016), but commissural visual pathways have yet to be investigated.

We found GF responses are invariant to looming stimulus azimuthal locations. This invariance is aligned with the GF's role in escape: the GF drives synchronized wing depression and leg extension in takeoff escapes regardless of stimulus position (Allen et al., 2006; Card and Dickinson, 2008; von Reyn et al., 2014). While our work here focuses on azimuthal invariance, recent investigations have identified anterior/posterior bias in VPN synapses onto looming responsive DN that confer directionality to escape trajectories (Dombrovski et al., 2023; Peek, 2018).

We found the illumination level of the contralateral eye modifies the processing of dynamic stimuli in the ipsilateral eye. Occluding the contralateral eye shifted ipsilateral and frontal tuning towards abrupt stimuli – the appearance of 10 deg disks or rapidly expanding disks (Fig. 3F,H). This may be similar to luminance gain modulation or compensatory plasticity occurring in vertebrates following occlusion of the contralateral eye (Ding and Sperling, 2006; Dougherty et al., 2021; Lunghi et al., 2011; Moradi and Heeger, 2009; Sato and Stryker, 2008). Our work suggests this modulation has differential effects on visual features – a large effect on angular velocity (LC4) but a minimal to small effect on angular size (LPLC2) encoding. Interestingly, vertebrate occlusion studies have also witnessed differential effects across visual feature pathways (Lunghi et al., 2013). The underlying mechanisms, however, are not well established. We believe future work in the *Drosophila* GF circuit will help shed light on these mechanisms.

## Supplementary Material

10.1242/jexbio.244790_sup1 Supplementary information
