# Supplementary material for: Azimuthal invariance to looming stimuli in the Drosophila giant fiber escape circuit
Source: J Exp Biol. 2023 Apr 20;226(8):jeb244790. doi: 10.1242/jeb.244790 (PMC10263144; doi:10.1242/jeb.244790)
Supplement: Supplementary information [file jexbio-226-244790-s1.pdf]

Table S1. Power analysis

Click here to download Table S1

Journal of Experimental Biology • Supplementary information
